# Studies on the synthesis, characterization, binding with DNA and activities of two *cis*-planaramineplatinum(II) complexes of the form: *cis*-PtL(NH_3_)Cl_2 _where L = 3-hydroxypyridine and 2,3-diaminopyridine

**DOI:** 10.1186/1472-6769-6-3

**Published:** 2006-03-13

**Authors:** Ahmed Abdullah, Fazlul Huq, Ashraf Chowdhury, Hasan Tayyem, Philip Beale, Keith Fisher

**Affiliations:** 1 School of Biomedical Sciences, Cumberland Campus, C42, The University of Sydney, East Street, PO Box 170, Lidcombe, NSW 1825, Australia; 2 RPAH, Missenden Road, Camperdown NSW, Australia; 3 School of Chemistry, F11, University of Sydney, NSW 2006, Australia

## Abstract

**Background:**

*Cis*-planaramineplatinum(II) complexes like their *trans *isomers are often found to be active against cancer cell lines. The present study deals with the synthesis, characterization and determination of activity of new *cis*-planaramineplatinum(II) complexes.

**Results:**

Two *cis*-planaramineplatinum(II) complexes: cis-(3-hydroxypyridine)(ammine)dichloroplatinum(II) (code named AH3) and *cis*-(2,3-diaminopyridine)(ammine)dichloroplatinum(II) (code named AH7) have been prepared and characterised based on elemental analyses, IR, Raman, mass and 1H NMR spectral measurements. The interactions of the compounds with pBR322 plasmid DNA have been investigated and their activity against ovarian cancer cell lines: A2780, A2780^cisR ^and A2780^ZD047R^have been determined. Like cisplatin, AH3 and AH7 are believed to form mainly monofunctional N7(G) and bifunctional intrastrand N7(G)N7(G) adducts with DNA, causing a local distortion of a DNA strand. As a result, gel mobility of the DNA changes. Both AH3 and AH7 are found to be less active than cisplatin against the three cell lines with AH3 being the more active compound of the two. The higher activity of AH3 is in line with its lower molar conductivity value corresponding to a lower degree of dissociation.

**Conclusion:**

The differences in activity of AH3, AH7 and cisplatin against the cell lines illustrate structure-activity relationship.

## Background

Although cisplatin is a widely used anticancer drug [1,2], it has a number of side effects and a limited spectrum of activity [3-7]. In an attempt to reduce toxicity and widen the spectrum of activity thousands of cisplatin analogues have been prepared by varying the nature of the leaving groups and the carrier ligands [8]. By manipulating the structure the leaving groups it has been possible to reduce toxicity (eg substitution of the more stable cyclobutanedicarboxylate for the two chlorides led to the development of carboplatin which produces substantially less nausea, vomiting and neurotoxicity but causes more of myelosuppression) and that of the carrier ligands, it has been possible to achieve a limited change in the spectrum of activity (eg oxaliplatin which has 1,2-diaminocyclohexane as the carrier ligands has been found to be active against colorectal cancer whilst cisplatin is not) [9-11]. In spite of the progress made, it is generally true to say that all cisplatin analogues have a similar spectrum of activity and develop cross resistance with cisplatin [8].

Currently attention is given to rule breaker platinum compounds with the idea that different nature of interaction with DNA may translate into a different spectrum of activity. One such class of compounds are *trans*-planaramineplatinum(II) complexes a number of which have been found to be quite active against both cisplatin-responsive and cisplatin-resistant cancer cell lines [12]. Often it is found that the *cis*-isomers of the compounds are also active. For example, ZD0D473 has been found to show significant antitumour activity against a number of cancer cell lines [13].

**Figure 1:**
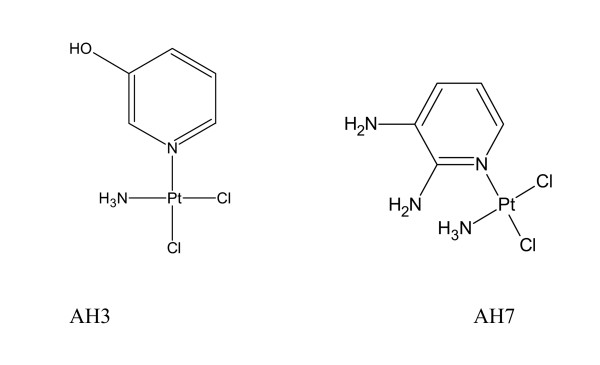
Structures of AH3 and AH7.

In our laboratory a number of *trans*-planaramineplatinum(II) complexes of the form: *trans*-PtCl_2_NH_3_L (L = 2-hydroxypyridine, 3-hydroxypyridine, imidazo [1,2-α]pyridine) have been prepared which have shown significant anticancer activity [14,15]. One of the compounds is twice as active as cisplatin against A2780^cisR ^cell line. The variations in activity of the compounds and conformational changes induced in pBR322 plasmid DNA illustrate structure-activity relationship. Later we reported on the synthesis and activity of some *cis*-planaramineplatinum(II) complexes as well [16]. In this paper, we report on the synthesis, binding with DNA and activity of two *cis*-planaramineplatinum(II) complexes of the form: *cis*-PtL(NH_3_)Cl_2 _where L = 3-hydroxypyridine and 2,3-diaminopyridine (Figure 1). Both the compounds are found to be less active than cisplatin against three ovarian cancer cell lines: A2780, A2780^cisR ^and A2780^ZD047R^.

## Results and discussion

### Characterisation

The compositions of AH3 and AH7 are given in Table 1.

**Table 1:** Composition of AH3 and AH7

	AH3		AH7	
	*Calculated %*	*Observed %*	*Calculated%*	*Observed %*

C	15.9	16.5 ± 0.4	15.3	15.1 ± 0.4
H	2.1	2.2 ± 0.4	2.6	2.3 ± 0.4
N	7.4	7.1 ± 0.4	14.3	13.8 ± 0.4
Cl	18.8	18.6 ± 0.3	18.1	18.3 ± 0.3
Pt	51.6	50.8 ± 1.0	49.8	49.9 ± 1.0

Significant difference between calculated and observed carbon contents for AH3 indicates that the compound contains some impurity possibly *cis*-bis(3-hydroxypyridine)dichloroplatinum(II).

### Molar conductivity

**Figure 2:**
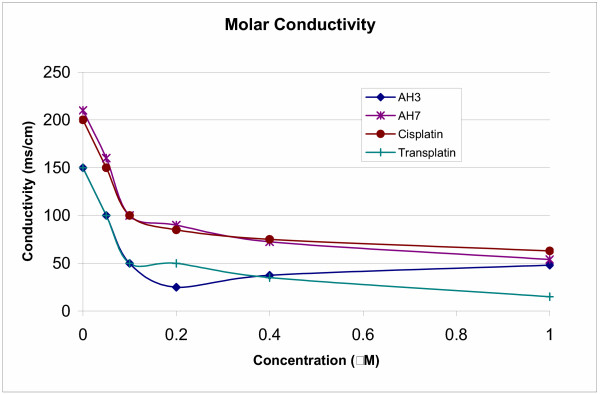
Plots of molar conductivities against concentrations as applied to AH3, AH7, cisplatin and transplatin.

Figure 2 gives plots of molar conductivities against concentrations as applied to AH3, AH7, cisplatin and transplatin.

The limiting values of molar conductivity (in ohm^-1 ^cm^2 ^mol^-1^) at zero concentration of AH3 and AH7 were found to be 150 and 210 respectively as against 200 for cisplatin and 150 for transplatin. These values are significantly lower than the expected value of about 280 for a 1:2 electrolyte, indicating that the degree of dissociation is about 54% in the case of AH3 and 75% in the case of AH7. The results suggest that AH3 may be crossing the cell membrane more readily than AH7 by passive diffusion. Since the degree of dissociation is significant, both the molecules may be crossing the cell membrane also by carrier-mediated transport (more so for AH7 than AH3). The lower molar conductivity values of AH3 and AH7 as compared to cisplatin suggests that the degree of dissociation of the compounds is less than that for cisplatin. This is in line with the presence of a bulkier planaramine ligand in AH3 and AH7 that introduces a greater steric hindrance. It is not however clear why transplatin is found to have a lower molar conductivity value than cisplatin. It should however be noted that 1:1 mixture of DMF and water does not truly represent biological fluids such as extracellular and intracellular fluids where the degree of dissociation is expected to be higher because of a higher water content.

### IR and Raman spectral analyses

The prominent bands observed in the IR and Raman spectra of AH3 and AH7 are given in Table 2.

**Table 2:** Prominent IR Raman spectral bands observed in AH3 and AH7 (ν cm^-1^)

	IR (cm^-1^)	Raman
AH3	3309 (s, NH and OH), 3062 (w, CH), 1649(w, NH), 1595 (s, 'aromatic ring'), 1444 (s, 'aromatic ring'), 1281 (s, OH), 1213 (w, CO), 1105 (w, CO), 1028 (ring stretch), 798 (m, CH), 687 (m, CH), 580 (w, Pt-N), 546 (m, Pt-N), 514 (m, Pt-N), 432 (m, pyridine ring)	3076 (m, NH), 2467 (m br, CH) 1603 (w, NH), 1030 (s, CH), 866 (m, CH), 664 (w, pyridine ring), 519 (w, Pt-N(NH_3_)), 330 (m, Pt-N(3-hydroxypyridine)), 243 (w, Pt-N(2-hydroxypyridine)), 122 (s, Pt-N), 81 (m, lattice)
AH7	3192 (s br, NH and CH stretch), 1614 (m, NH bending), 1539 (s, aromatic ring stretch), 1371 (m, CH bending), 1302 (s, ring in-plane deformation), 1211 (m, CH in-plane bending of heterocyclic ring), 777 (w d, ring out-of-plane deformation), 496 (w, Pt-N stretch)	3562 (w, NH stretch), 3314 (s, NH stretch), 2806 (m, CH stretch), 2361 (m, ring stretch), 1537 (m, NH bending), 1030 (s, CH in-plane bending), 797 (m, ring out-of-plane deformation), 346 (m, Pt-Cl stretch), 95 (m, ring bending)

The letters 's', 'm', 'w', 'd' and 'br' stand for 'strong', 'medium', 'weak', 'doublet' and 'broad' respectively.

## AH3

### IR

The broad band at 3309 cm^-1 ^is believed due to O-H and N-H stretching vibrations. The band at 3062 cm^-1 ^is believed to be due to C-H stretch. The bands at 1649 and 1595 cm^-1 ^are due to N-H bending vibrations whereas that at 1444 cm^-1 ^is due to C-H bending vibration. The band at 1496 cm^-1 ^is due to 'aromatic' ring stretch. The band at 1281 cm^-1 ^is due to O-H bending vibration. The bands at 1213 and 1105 cm^-1 ^are due to C-O stretching vibrations. The band at 1028 cm^-1 ^is due to ring stretch. The bands at 687 and 798 cm^-1 ^are due to C-H out of plane bending vibrations. The band at 879 cm^-1 ^is believed due to N-H wagging. The bands at 580, 546 and 432 cm^-1 ^are due to Pt-N stretching vibrations.

### Raman

The band at 3076 cm^-1 ^is believed due to N-H stretching vibration whereas that at 2467 cm^-1 ^and is believed to be due to C-H stretching vibration. The band at 2307 cm^-1 ^is believed to be associated with the vibration of 2-hydroxypyridine ring. The band at 1603 cm^-1 ^is due to N-H bending vibration whereas the bands at 1067, 1030 and 664 cm^-1 ^are due to C-H in plane bending of the heterocyclic ring. The band at 866 cm^-1 ^is due to N-H wagging. The band at 519 cm^-1 ^is due to Pt-N(NH_3_) stretching vibration and that at 330 cm^-1 ^is due to Pt-Cl stretching vibration. The band at 243 cm^-1 ^is due to Pt-N(3-hydroxypyridine) stretch whereas that at 122 cm^-1 ^is due Pt-N bending vibration. The band at 76 cm^-1 ^is associated with the lattice mode.

## AH7

The band at 3192 is due to N-H and C-H stretching vibrations. The band at 1614 cm^-1 ^is due to N-H bending vibration whereas that at 1539 cm^-1 ^is due to aromatic ring stretch. The band at 1371 is due to C-H bending vibration. The band at 1302 cm^-1 ^is due to in-plane deformation of the heterocyclic ring whereas that at 777 cm^-1 ^is due to its out-of-pane deformation. The band at 496 cm^-1 ^is due to Pt-N stretching vibration.

### Raman

The band at 3562 and 3314 are believed due to N-H stretching vibration whereas that 2806 is believed to be due to C-H stretching vibration. The band at 2361 cm^-1 ^is believed to be associated with the vibration of pyridine ring. The band at 1537 cm^-1 ^is due to N-H bending vibration whereas the band at 1030 cm^-1 ^is due to C-H in-plane bending of the heterocyclic ring. The band at 346 cm^-1 ^is due Pt-Cl stretch. The band at 95 cm^-1 ^is due to bending vibration of the pyridine ring.

#### Mass 1H and NMR spectral analyses

The prominent peaks observed in the EIS mass and ^1^H NMR spectra of AH3 and AH7 are given in table 3.

**Table 3:** Prominent peaks observed in the ESI mass and ^1^H NMR spectra of AH3 and AH7

	ESI Mass	^1^H NMR
AH3 MM = 378.1	EIS-MS (DMF) (ClPt-μ-(NH)Pt(Cl)(3-hydroxypyridine)-μ-(NH)Pt(3-hydroxypyridine) = 876 (0.57); (NH_2_)_2_Pt-μ-(NH)Pt(pyridine) = 516 (0.98); (M - OH - 4H) = 357 (0.11); (M - NH_3 _- 2Cl + 3-hydroxypyridine - H) = 384 (1.00)	^1 ^H NMR DMSO δ ppm: 10.80 (s br, due to OH); 8.42 (d, due to CH ortho); 8.14 (d, due to CH ortho); 8.03 (d, due to CH para); 8.02 (quartet, due to CH meta); 4.30 (s, due to NH-Pt); 3.86 (s, NH-Pt); 3.34 (s, due to water); 2.50 (due to DMSO)
AH7 MM = 392.1	EIS-MS (DMF) (PtCl(2,3-diaminopyridine) = 516 (0.36); (M - Cl + 2,3-diaminopyridine) = 438 (1.00); (M - H) = 377 (0.09); (M - H -NH_3_) = 360; (M - Cl - NH_3 _- H) = 324 (0.66); (M - Cl - (2,3-diaminoyridine) + NH_3_) = 265 (0.31)).	^1^H NMR DMSO δ ppm: 7.30 (due to CH); 7.20 (due to CH); 6.90 (due to CH); 4.02 (s; NH-Pt); 3.34 (d, due to water); 2.70 (NH), 2.50 (due to DMSO), 2.40 (s, NH))

The letters 's', 'm', 'w', 'd' and 'br' stand for 'strong', 'medium', 'weak', 'doublet' and 'broad' respectively.

## Mass

### AH3

The mass spectrum of AH3 has a peak with m/z = 876 corresponding to (ClPt-μ-(NH)Pt(Cl)(3-hydroxypyridine)-μ-(NH)Pt(3-hydroxypyridine) that is believed to be formed in situ from joining of the fragments produced from AH3. The peak with m/z = 516 corresponds to (NH_2_)_2_Pt-μ-(NH)Pt(pyridine) that is also believed to be formed in situ from joining of the fragments produced from AH3. The peak with m/z = 357 corresponds to (M - OH - 4H) and that with m/z = 384 corresponds to (M - NH_3 _- 2Cl + 3-hydroxypyridine - H).

### AH7

The mass spectrum of AH3 has a peak with m/z = 516 corresponding to (PtCl(2,3-diaminopyridine) that is believed to be formed in situ from joining of the fragments produced from AH3. The peak with m/z = 438 corresponds to (M - Cl + 2,3-diaminopyridine) that is also believed to be formed in situ from joining of the fragments produced from AH3. The peak with m/z = 360 corresponds to (M - H -NH_3_) and that with m/z = 265 corresponds to (M - Cl - (2,3-diaminoyridine) + NH_3_).

### ^1^H NMR

#### AH3 [See Additional file 1]

The resonance at δ = 10.80 ppm is due to OH proton, that at 8.42 ppm is due to CH that lies in between the nitrogen centre and the carbon to which OH group is attached. The resonance at δ = 8.14 ppm is due to CH at the other ortho position and that at δ = 8.03 ppm is due to CH at the para position. The resonance at δ = 8.02 ppm is due to CH at the meta position. The resonances at δ = 4.30 and 3.86 ppm are due to NH-Pt. The resonance δ = 3.34 ppm is due to water and that 2.50 is due to DMSO.

#### AH7 [See Additional file 2]

The resonances at δ = 7.30, 7.20 and 6.90 ppm are due to CH. The resonance at 4.02 ppm is due to NH-Pt, that at 3.34 is due to water and that at 2.50 is due to DMSO. The resonances at δ = 2.70 and 2.40 ppm are due to NH.

#### Interaction with pBR322 plasmid DNA

**Figure 3:**
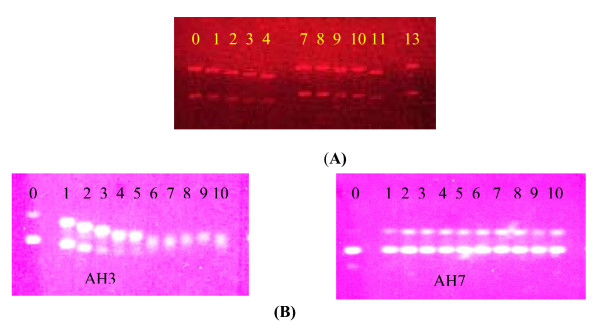
**(A)**: Interaction of pBR322 plasmid DNA with increasing concentrations of cisplatin (lanes 0–4: 1: 1 μM; 2: 2.5 μM; 3: 5 μM; 4: 10 μM.) and transplatin (lanes 7–13: 7: 0 8: 1 μM; 9: 2.5 μM; 10: 5 μM; 11: 10 μM, 13: 0.) ranging from 1 to 10 μM. **(B)**: Interaction between pBR322 plasmid DNA and increasing concentrations of AH3 and AH7 ranging from 5 μM to 50 μM: (Lanes 0 to 10: lane 0: untreated plasmid, 1:5 μM; 2: 10 μM; 3: 15 μM; 4: 20 μM; 5: 25 μM; 6: 30 μM; 7: 35 μM; 8: 40 μM; 9: 45 μM; 10: 50 μM).

Figure 3 gives the electrophoretograms applying to the interaction of pBR322 plasmid DNA with cisplatin, transplatin, AH3 and AH7 at concentrations ranging from 1 μM to 10 μM in the case of cisplatin and transplatin and 5 μM to 50 μM in the case of AH3 and AH7.

Figure 3 here

As pBR322 plasmid DNA, which is found to be a mixture of mainly supercoiled form I and a small amount of singly nicked circular form II, is interacted with increasing concentrations of AH3, the mobility of both form I and form II bands changes such that the two bands co-migrate for the concentrations of AH3 ranging from 30 to 40 μM above which the two bands partially separate. There is also a decrease in intensity of the bands at higher concentrations of AH3. The change in mobility of the form I band is due to a change in its conformation brought about by covalent binding of AH3 with the DNA. AH3 is expected to form mainly monofunctional Pt-G and intrastrand bifunctional Pt(GG) adducts with DNA, the latter causing the bending of a DNA strand mainly at the binding site. The decrease in intensity of the bands is due to DNA damage brought about possibly by the stacking interaction between 3-hydroxypyridine ligand in AH3 and nucleobases in DNA. The sharp increase in intensity of form II band at low concentrations of AH3 as compared to that in untreated DNA indicates the conversion of some of form I DNA to form II DNA due catalyzed by AH3. When pBR322 plasmid DNA is interacted with increasing concentrations of AH7 also, there is an initial increase in intensity of the form II band at low concentrations of the compound, indicating the conversion of some of form I DNA to form II DNA as in the case of AH3. The mobility of both forms I and II bands appear to remain unchanged with the change in concentration of AH7. The results suggest that AH7 is less able to cause conformational change in pBR322 plasmid DNA than AH3. It may be noted that the presence of two amine groups in 2,3-diaminopyridine makes it slightly bulkier than 3-hydroxypyridine. As a result, steric constraint hindering access to platinum would be greater in AH7 than AH3. As discussed earlier, 2,3-diaminopyridine and 3-hydroxypyridine are also expected to differ in their polarity. This factor too may have some influence on the reactivity of AH3 and AH7 and their binding with DNA.

#### BamH1 digestion

**Figure 4:**
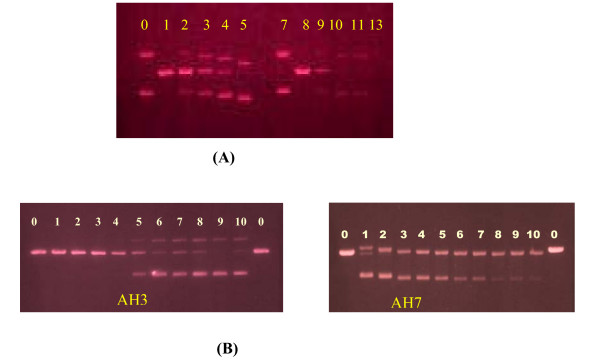
**(A)**: Electrophoretogram for the incubated mixtures of pBR322 plasmid DNA and varying concentrations of cisplatin (lanes 0–5: 0: untreated plasmid; 1: 0, 2: 1 μM; 3: 2.5 μM; 4: 5 μM; 5: 10 μM) and transplatin (lanes 7–13: 7: untreated plasmid; 8: 0, 9: 1 μM; 10: 2.5 μM; 11: 5 μM; 12: 10 μM) **(B)**: Electrophoretogram for the incubated mixtures of pBR322 plasmid DNA and varying concentrations of: AH3 and AH7 (lane 0: untreated and undigested DNA, lane 1: 5 μM, lane 2: 10, lane 3: 15 μM, lane 4: 20 μM, lane 5: 25 μM, lane 6: 30 μM, lane 7: 35 μM, lane 8: 40 μM, lane 9: 45 μM, lane 10: 50 μM) followed by digestion with BamH1. Lane B applies to untreated but digested pBR322 plasmid DNA.

Figure 4 shows the electrophoretograms for the incubated mixtures of pBR322 plasmid DNA and varying concentrations of cisplatin and transplatin, AH3 and AH7, followed by BamH1 digestion. For cisplatin and transplatin, concentration ranges from 0 to 10 μM and for AH3 and AH7 it ranges from 0 to 50 μM. In the absence of platinum compounds, only the linear form III band is observed, indicating that all of the pBR322 plasmid DNA is doubly nicked by BamH1 at the specific GG site. At low concentrations of AH3 (0 to 20 μM), only form III band is observed whereas at higher concentrations of the compound (25 μM to 50 μM) a mixture of forms I, II and III bands are observed. At the lowest concentration of AH7 (5 μM), a mixture of forms I, II and III is observed and at higher concentrations (10 μM or greater), a mixture of forms I and II is observed. The results suggest that AH7 is able to cause a greater conformational change to the DNA than AH3, but as noted earlier, AH3 causes a greater change in mobility of pBR322 plasmid DNA than AH7.

A mixture of forms I, II and III bands are observed for all concentrations of cisplatin ranging from 1 to 10 μM with forms I and II bands being the faintest at 1 μM cisplatin and form III band being the faintest at 10 μM cisplatin. The results indicate increasing prevention of BamH1 digestion with the increase in concentration of cisplatin. This is due to conformational change in the DNA brought about by the covalent binding of cisplatin with the DNA (mainly intrastrand GG). A mixture of forms I, II and III was also observed for all concentrations of transplatin indicating that transplatin is also able to prevent BamH1 digestion. However, the relative intensities of the three bands suggest that transplatin is less able to prevent BamH1 digestion than cisplatin. Like cisplatin transplatin can form monofunctional adducts but unlike cisplatin (which forms intrastrand bifunctional adducts), transplatin is more likely to form interstrand bifunctional adducts. The results suggest that the formation of interstrand adducts also brings about sufficient conformational change in DNA (so as to prevent BamH1 digestion) mainly due to mismatch between interbase distance and that between the two *trans *arms of transplatin. The progressive decrease in intensity of the bands from lanes 9 to 13 appears to be artefact as the band applying to untreated and undigested DNA (lane 13) could not be seen.

#### Cytotoxicity

Table 4 gives the IC_50 _values of AH3, AH7 and cisplatin against the cell lines: A2780, A2780^cisR ^and A2780^ZD047R^.

**Table 4:** IC_50 _value and resistance factors for AH3, AH7 and cisplatin against the cell lines: A2780, A2780^cisR ^and A2780^ZD047R^.

	A2780	A2780-cisR	IC_50_A2780^cisR^/IC_50_A2780	A2780^ZD0473R^
	IC_50_	IC_50_	RF	IC_50_

AH3	3.8 ± 1.0	6.9 ± 1.0	1.8	7.5 ± 1.0
AH7	11.7 ± 1.4	8.5 ± 1.3	0.7	10.6 ± 1.0
Cisplatin	1.1 ± 0.4	2.3 ± 0.4	2.1	2.2 ± 0.4

The letters 's', 'm', 'w', 'd' and 'br' stand for 'strong', 'medium', 'weak', 'doublet' and 'broad' respectively.

It can be seen that both AH3 and AH7 are significantly less active than cisplatin against the cell lines: A2780, A2780^cisR ^and A2780^ZD0473R^. It should however be noted the IC_50 _value for cisplatin found in the present study for the cell line A2780 is significantly higher than the reported value [24]. This difference may be the result of some problems faced in growing A2780 cells in the present study. It is thus possible that the underestimation of activity may apply to AH3 and AH7 so that the actual IC_50 _value of AH3 and AH7 for the cell line A2780 could be significantly lower than the value found.

The other point to note is that although AH3 and AH7 are less active than cisplatin, the change in activity of the compound in going from cisplatin-responsive cell line: A2780 to the resistant cell lines: A2780^cisR ^and A2780^ZD0473R ^is less marked than that for cisplatin, The results suggest that at the level of their activity the compounds have been able to overcome mechanisms of resistance operating in A2780^cisR ^and A2780^ZD0473R ^cell lines. As stated earlier, like cisplatin AH3 and AH7 are expected to form monofunctional and intrastrand bifunctional adducts with DNA. It is possible that the presence of a bulky planaramine ligand makes AH3 and AH7 much less reactive than cisplatin. Also, some AH3 and AH7 molecules may simply undergo intercalation with DNA or while binding covalently with nucleobases in DNA may undergo stacking interaction. Damage to pBR322 plasmid DNA caused by AH3 and AH7 (discussed earlier) may be due to intercalation.

AH3 is found to be more active than AH7 against the three cell lines. It should be recalled that molar conductivity value of AH7 is found to be higher than that for AH3. The presence of two amino groups in 2,3-diaminopyridine ligand make AH7 more ionisable than AH3 in which the planaramine is 3-hydroxypridine. It is possible that both the cell uptake and level of binding with DNA will be for AH7 than AH3 which would explain the lower activity of AH7 than AH3.

## Conclusion

Two *cis*-planaramineplatinum(II) complexes: *cis*-(3-hydroxypyridine)(ammine)dichloroplatinum(II) (code named AH3) and *cis*-(2,3-diaminopyridine)(ammine)dichloroplatinum(II) (code named AH7) have prepared and characterised based on elemental analyses, IR, Raman, mass and ^1^H NMR spectral measurements. The interaction of the compounds with pBR322 plasmid DNA has been studied and the activity of the compound against ovarian cell lines: A2780, A2780^cisR ^and A2780^ZD0473R ^have also been determined where cisplatin has been used as the reference. Like cisplatin, AH3 and AH7 are expected to form monofunctional and intrastrand bifunctional adducts with DNA, but the compounds are found to cause a weaker unwinding of supercoiled form I pBR322 plasmid DNA and the prevention of BamH1 digestion. The lower activity of AH3 and AH7 as compared to cisplatin is believed to be due to their reduced ability to bind with DNA because of a greater steric constraint introduced by bulkier planaramine ligands. AH3 is found to be more active than AH7 against the three cell lines. It is believed that the presence of two amino groups in 2,3-diaminopyridine ligand make AH7 more ionisable than AH3 in which the planaramine is 3-hydroxypridine. It is possible that both the cell uptake and level of binding with DNA will be for AH7 than AH3 which would explain the lower activity of AH7 than AH3. The determination of cell uptake and the level of binding with DNA would indicate whether the idea is true or not.

## Methods

### Materials

Potassium tetrachloroplatinate (K_2 _[PtCl_4_]), 2,3-diaminopyridine [C_7_H_7_N_2_], N, N-dimethylformamide [DMF] [C_3_H_7_NO], 3-hydroxypyridine, cisplatin and transplatin were obtained from Sigma Aldrich Chemical Company Milwaukee USA; acetone [(CH_3_)_2_CO] and silver nitrate [AgNO_3_] were obtained from Ajax Chemicals Auburn NSW Australia; methanol [CH_3_OH] and ethanol [C_2_H_5_OH] were obtained from Merck Pty. Limited Kilsyth VIC Australia. pBR322 plasmid DNA was obtained from ICN Biochemicals Ohio USA.

### Syntheses

*cis*-planaramineplatinum(II) complexes: *cis*-(3-hydroxypyridine)(ammine)dichloroplatinum(II) (code named AH3) and *cis*-(2,3-diaminopyridine)(ammine)dichloroplatinum(II) (code named AH7) have synthesized according to modified Dhara [17] method.

### *cis*-Pt(NH_3_)(3-hydroxypyridine)Cl_2 _(AH3)

415 mg (1 mmol) of K_2 _[PtCl_4_] was dissolved in 10 mL of milli Q (mQ) water to which was added 2 g (about 12 mmol) of KI. The mixture was stirred at room temperature for 5 min. 0.14 mL (1 mmol) of 1:1 aqueous solution of ammonia was added to the mixture with stirring that was kept in ice for about 30 min. The yellow precipitate of *cis*-Pt(NH_3_)I_3 _formed immediately after the addition of ammonia solution. 95 mg (1 mmol) of 3-hydroxypyridine, dissolved in 5 mL of mQ water by sonification, was added with stirring to the mixture that was kept in ice. The colour of the precipitate changed from yellow to brown after about 15 min at the end of which the precipitate of *cis*-Pt(3-hydroxypyridine)(NH_3_)I_2 _was collected by filtration, washed with ice-cold water and air-dried. [The time was found to be critical as longer period resulted in the formation of some *cis*-bis[(3-hydroxypyridine)]I_2_Pt(II).] The mass of the precipitate was 301 mg (0.5 mmol). The precipitate was suspended in 5 mL of mQ water to which was added 168 mg (0.99 mmol) of AgNO_3 _with stirring in the dark at room temperature. Stirring was continued for 3 h. The mixture was centrifuged to collect the yellow supernatant to which was added 80 mg (1.05 mmol) of KCl with stirring at room temperature. Stirring was continued for 24 h. The yellow precipitate of *cis*-Pt(3-hydroxypyridine)(NH_3_)Cl_2 _was collected by filtration, washed with mQ water and air-dried. The weight of the final product was 140 mg (0.4 mmol) corresponding to 40% yield. It was characterized by elemental analyses, IR, Raman, mass and ^1^H NMR spectral studies.

### cis-(2,3-diaminopyridine)(ammine)dichloroplatinum(II) (AH7)

415 mg (1 mmol) of K_2 _[PtCl_4_] was dissolved in 20 mL of mQ water to was added 4 g (about 24 mmol) of KI. The mixture was stirred at room temperature for 5 min. 0.14 mL (1 mmol) of 1:1 aqueous solution of ammonia was added to the mixture with stirring that was continued for about 30 min. 109 mg (1 mmol) of 2,3-diaminopyridine was added with stirring to the mixture at room temperature. Dark green precipitate of *cis*-Pt(2,3-diaminopyridine)(NH_3_)I_2 _formed immediately. Stirring was continued for 24 h at room temperature. The precipitate was collected by filtration, washed with ice-cold water and ethanol, and air-dried. The mass of the precipitate was 454 mg (0.8 mmol). The precipitate was suspended in 5 mL of mQ water to which was added 266 mg (1.57 mmol) of AgNO_3 _with stirring in the dark at room temperature. Stirring was continued for 10 h. The mixture was centrifuged to collect the black supernatant to which was added 125 mg (1.68 mmol) of KCl with stirring at room temperature that was continued for 24 h. The black precipitate of *cis*-Pt(2,3-diaminopyridine)(NH_3_)Cl_2 _was collected by filtration, washed with ice cold water and air-dried. The weight of the final product was 40 mg (0.1 mmol) corresponding to 10% yield. It was characterized by elemental analyses, IR, Raman, mass and ^1^H NMR spectral studies.

### Characterization

C, H, N and Cl were determined using the facility at the Australian National University. Platinum was determined by graphite furnace atomic absorption spectroscopy (AAS) using the Varian Spectraa-20 Atomic Absorption Spectrophotometer. Infrared spectra were collected using a Bruker IFS66 spectrometer equipped with a Spectra-Tech Diffuse Reflectance Accessory (DRA), an air-cooled DTGS detector, a KBr beamsplitter with a spectral range of 4000 to 400 cm^-1^. The instrument was run under a vacuum during spectral acquisition. Spectra were recorded at a resolution of 4 cm^-1^, with the co-addition of 128 scans and a Blackman-Harris 3-Term apodisation function was applied. Prior to analysis the samples were mixed, and lightly ground, with finely ground spectroscopic grade KBr. The spectra were then manipulated using the Kubelka-Munk mathematical function in the OPUS™ software to convert the spectra from reflectance into absorbance. Raman spectra were collected using a Bruker RFS100 Raman spectrometer equipped with an air cooled Nd:YAG laser emitting at a wavelength of 1064 nm, and a liquid nitrogen cooled germanium detector with an extended spectral band range of 3500 to 50 cm^-1^. 180° sampling geometry was employed. Spectra were recorded at a resolution of 4 cm^-1^, with the co-addition of 100 scans at a laser power of 0.065 mW. A Blackman-Harris 4-Term apodisation function was applied and the spectra were not corrected for instrument response. To obtain mass spectra, solution of AH3 and AH7, made in 10% DMF and 90% methanol, were sprayed into a Finnigan LCQ ion trap mass spectrometer in which fragmentation was produced by a high energy electron beam bombardment. ^1^H NMR spectra of AH3 and AH7 were recorded in dimethylsulfoxide-d_6 _(DMSO-d_6_) solution in a Bruker AVANCE DPX 400 spectrometer. Spectra were referenced to internal solvent residues and were recorded at 300 K (± 1 K).

### Molar conductivity

The molar conductivity values for AH3, AH7, cisplatin and transplatin were determined using PW9506 digital conductivity meter available at the School of Chemistry, University of Sydney. The compounds were dissolved in 1:1 mixture of DMF and water. The measurements were done at concentrations: 1 mM, 0.5 mM, 0.2 mM and 0.05 mM. The molar conductivity (Λ_m_) was calculated as Λ_m_= k/c where k is specific conductivity and c is the concentration [18]. The molar conductivity values were plotted against concentration and the curve was extrapolated to zero concentration to obtain the limiting value.

### Interaction with pBR322 plasmid DNA

Interaction of AH3, AH7 and cisplatin with pBR322 plasmid DNA was studied by agarose gel electrophoresis. The method used was a modification of that described by Stellwagen [19]. Briefly, solutions of pBR322 plasmid DNA (at concentration 0.5 μg mL^-1^) were incubated with increasing concentrations of compounds ranging from 4 μM to 50 μM in a shaking water bath at 37°C for 4 h, following which 16 μL aliquots of drug-DNA mixtures containing 0.6 μg of DNA was loaded onto the 1% gel and electrophoresis was carried under TAE buffer for 2 h at 5 V cm^-1^. The gel was stained in the same buffer containing ethidium bromide (0.5 mg mL^-1^) and visualised under UV light using the Bio-Rad Trans illuminator IEC 1010. The illuminated gel was photographed with a Polaroid camera (a red filter and Polaroid type of film was used).

### BamH1 digestion

BamH1 is known to recognize the sequence G/GATCC and hydrolyse the phosphodiester bond between adjacent guanine sites [20]. BamH1 contains a single restriction site for pBR322 plasmid DNA and is thus able to convert the DNA from supercoiled form I and singly nicked circular form II to linear form III DNA [21]. In this experiment, a same set of drug-DNA mixtures as that described previously, was first incubated for 4 h in a shaking water bath at 37°C and then subjected to BamH1 (10 units μL^-1^) digestion. To each 20 μL of incubated drug-DNA mixtures were added 3 μL of 10× digestion SB buffer followed by the addition of 0.2 μL BamH1 (2 units). The mixtures were left in a shaking water bath at 37°C for 1 h at the end of which the reaction was terminated by rapid cooling. The gel was subsequently stained with ethidium bromide, visualized under UV light and photographed as described previously.

## List of abbreviations

AH6: *cis*-bis(imidazo(1,2-α)pyridine)dichloroplatinum(II). Cisplatin: *cis*-dichlorodiamminplatinum(II). Transplatin: *trans*-dichlorodiamminplatinum(II). 1×TAE buffer: 0.05 M Tris base + 0.05 M glacial acetic acid + 1 mM EDTA, pH = 8.0. DMSO: Dimethyl sulfoxide. AAS: Atomic absorption spectrophotometry. A: Adenine. G: Guanine

## Authors' contributions

AA carried out all experiments described. AC helped in recording IR spectral data. HT helped with molar conductivity measurements. PB assisted in cell culture study. KF recorded the mass spectra. FH conceived the project and participated in the design and coordination of the study.

## Supplementary Material

Additional File 1 ^1^H NMR spectrum of AH3. This figure gives the ^1^H NMR spectrum of AH3. Click here for file

Additional File 2 ^1^H NMR spectrum of AH7. This figure gives the ^1^H NMR spectrum of AH7. Click here for file
