# Supplementary material for: Studies on the synthesis, characterization, binding with DNA and activities of two cis-planaramineplatinum(II) complexes of the form: cis-PtL(NH3)Cl2 where L = 3-hydroxypyridine and 2,3-diaminopyridine
Source: BMC Chem Biol. 2006 Mar 13;6:3. doi: 10.1186/1472-6769-6-3 (PMC1431574; doi:10.1186/1472-6769-6-3)

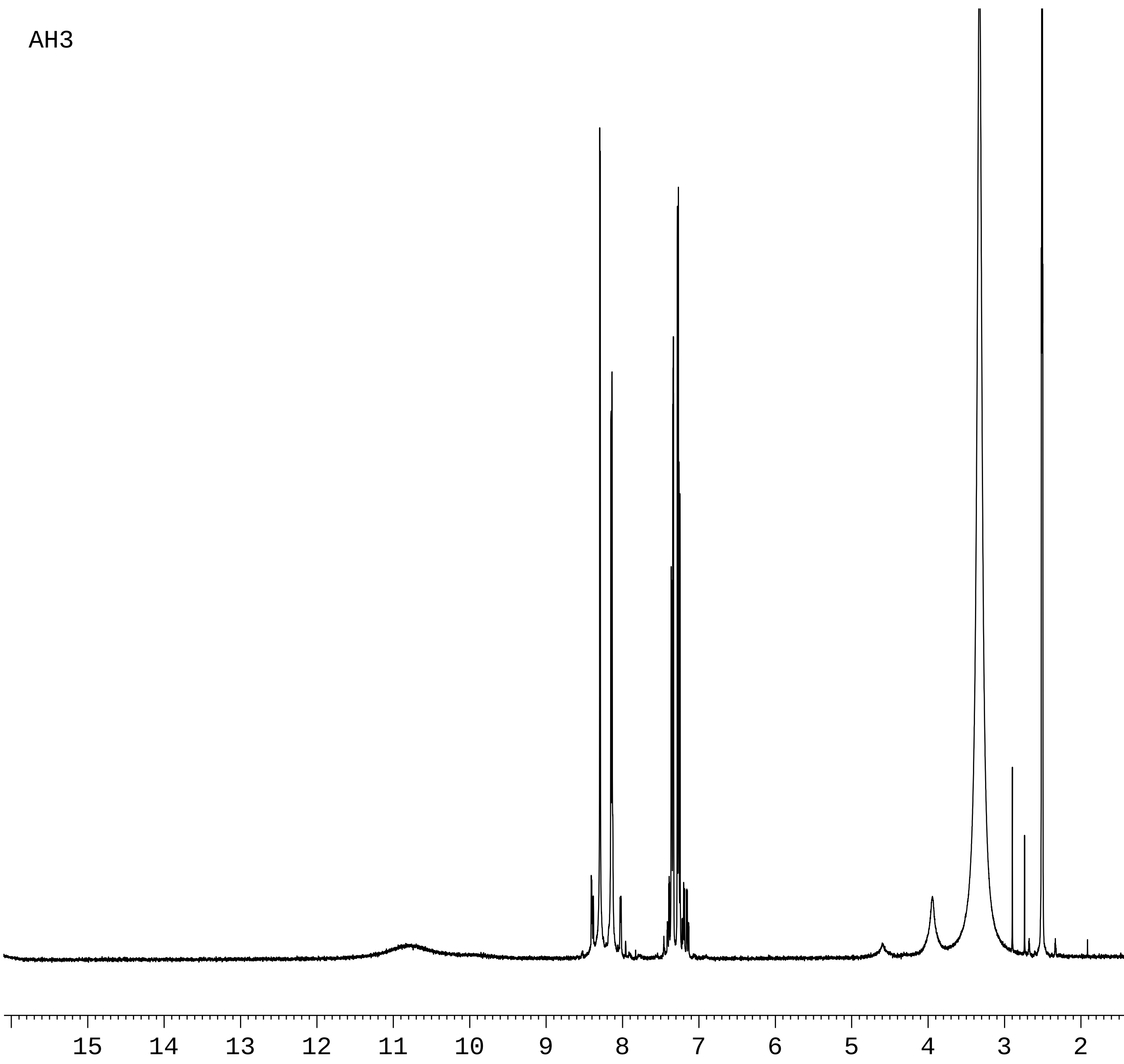
AH3
15 14 13 12 11 10 9 8 7 6 5 4 3 2

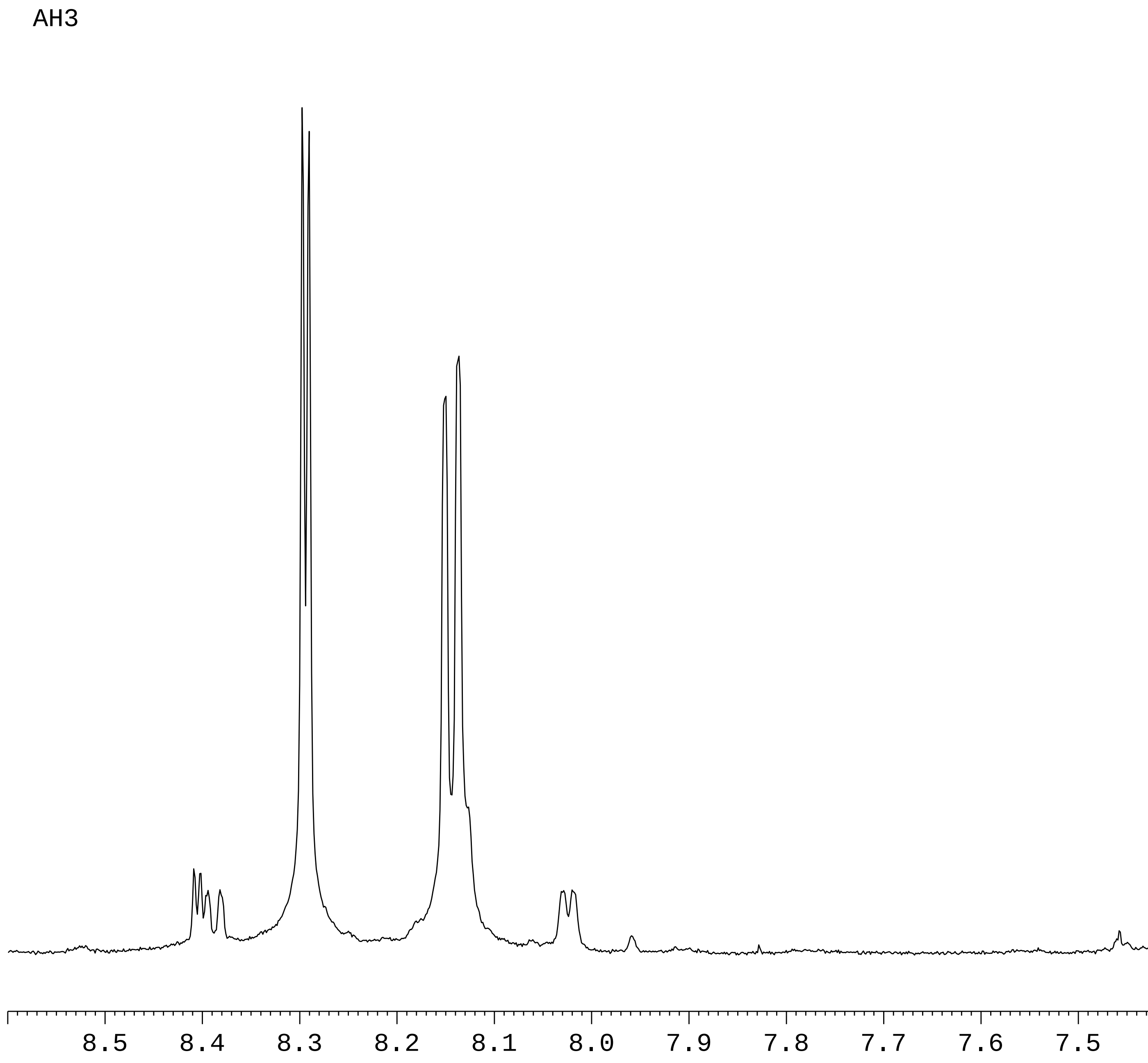
AH3
8.5
8.4
8.3
8.2
8.1
8.0
7.9
7.8
7.7
7.6
7.5

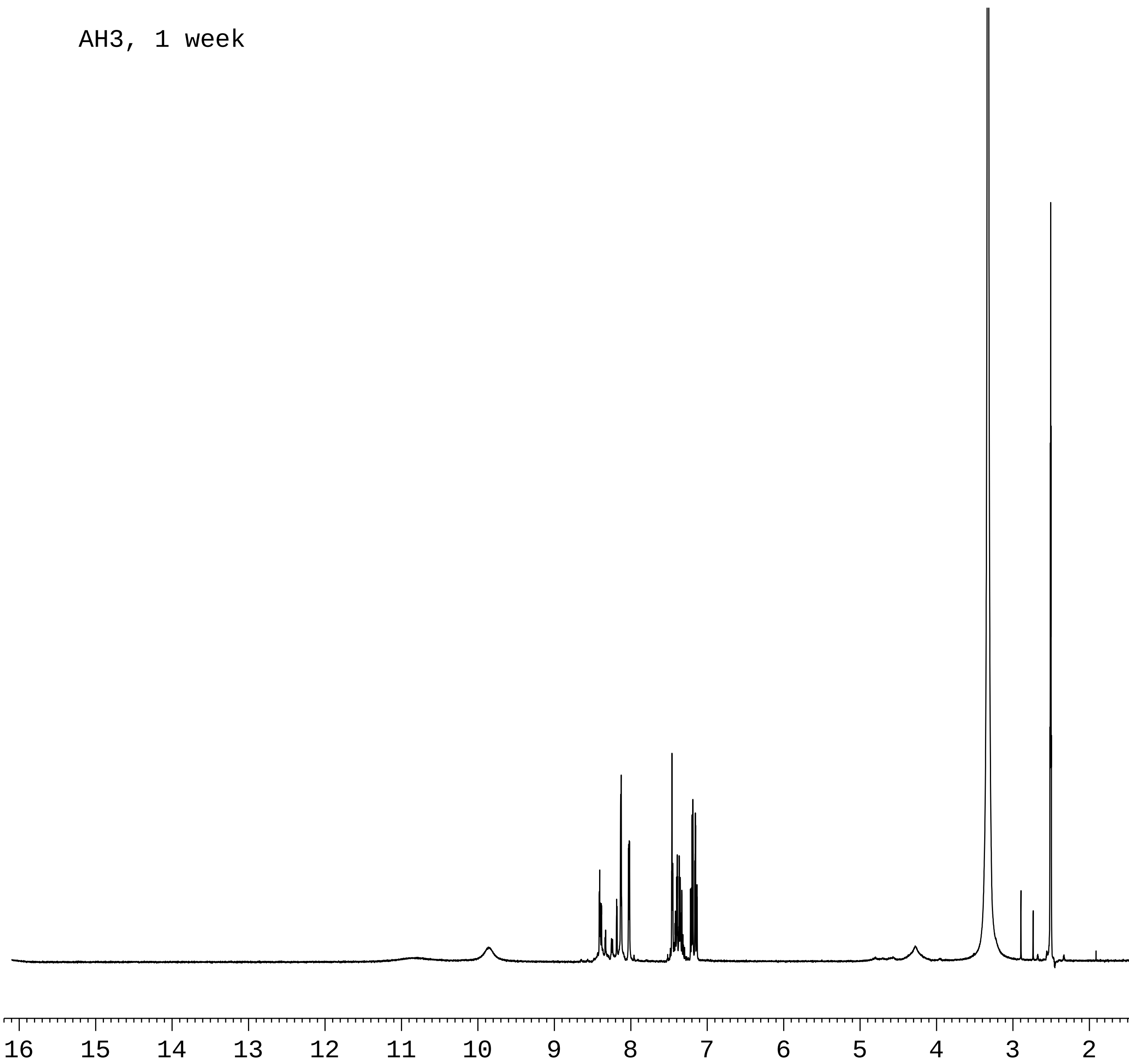
AH3, 1 week
16 15 14 13 12 11 10 9 8 7 6 5 4 3 2

AH3, 1 week

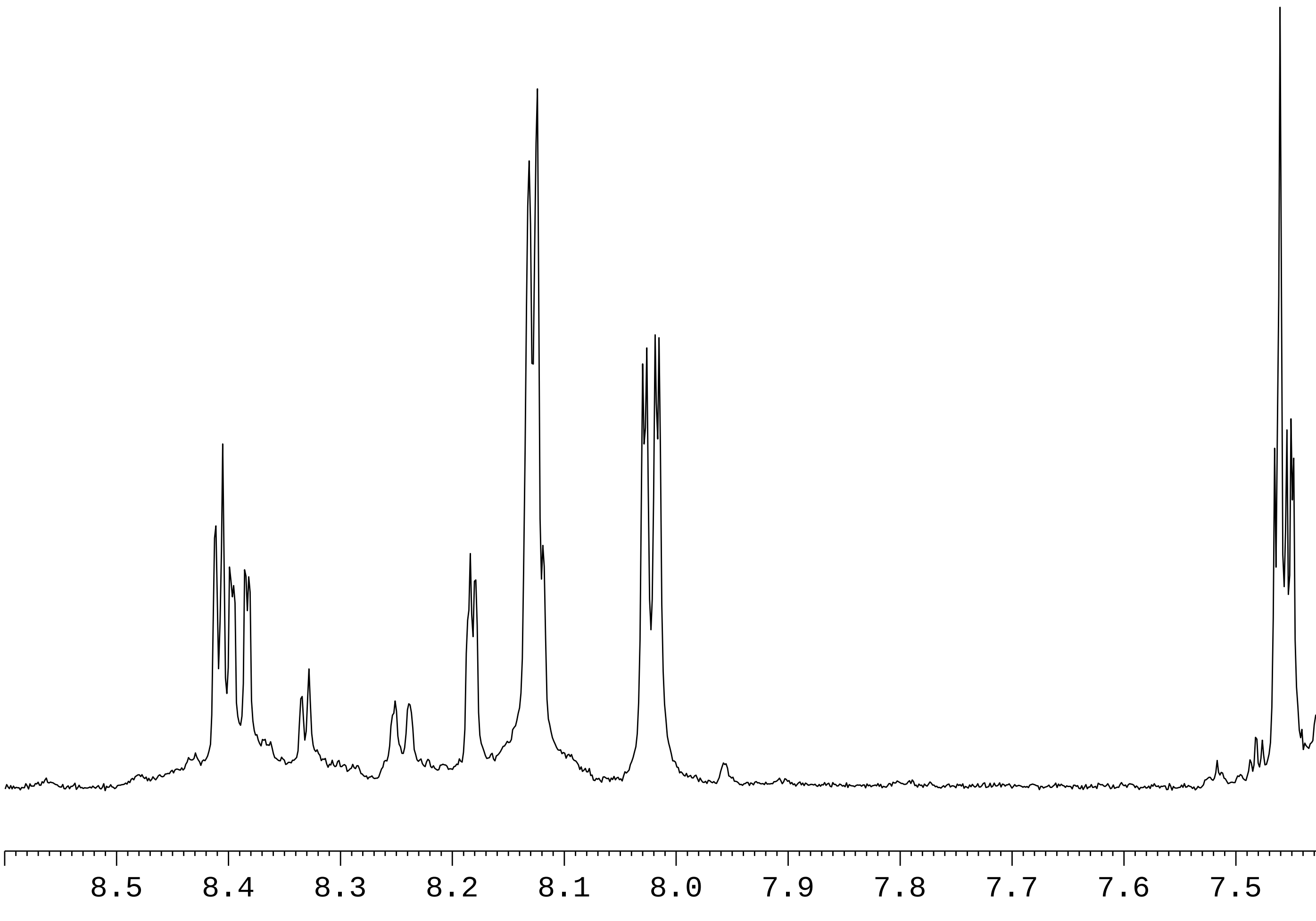

Supplement: Supplementary file 1 [file 1472-6769-6-3-S1.DOC]
