# Supplementary material for: Studies on the synthesis, characterization, binding with DNA and activities of two cis-planaramineplatinum(II) complexes of the form: cis-PtL(NH3)Cl2 where L = 3-hydroxypyridine and 2,3-diaminopyridine
Source: BMC Chem Biol. 2006 Mar 13;6:3. doi: 10.1186/1472-6769-6-3 (PMC1431574; doi:10.1186/1472-6769-6-3)
Supplement: Supplementary file 2 [file 1472-6769-6-3-S2.DOC]

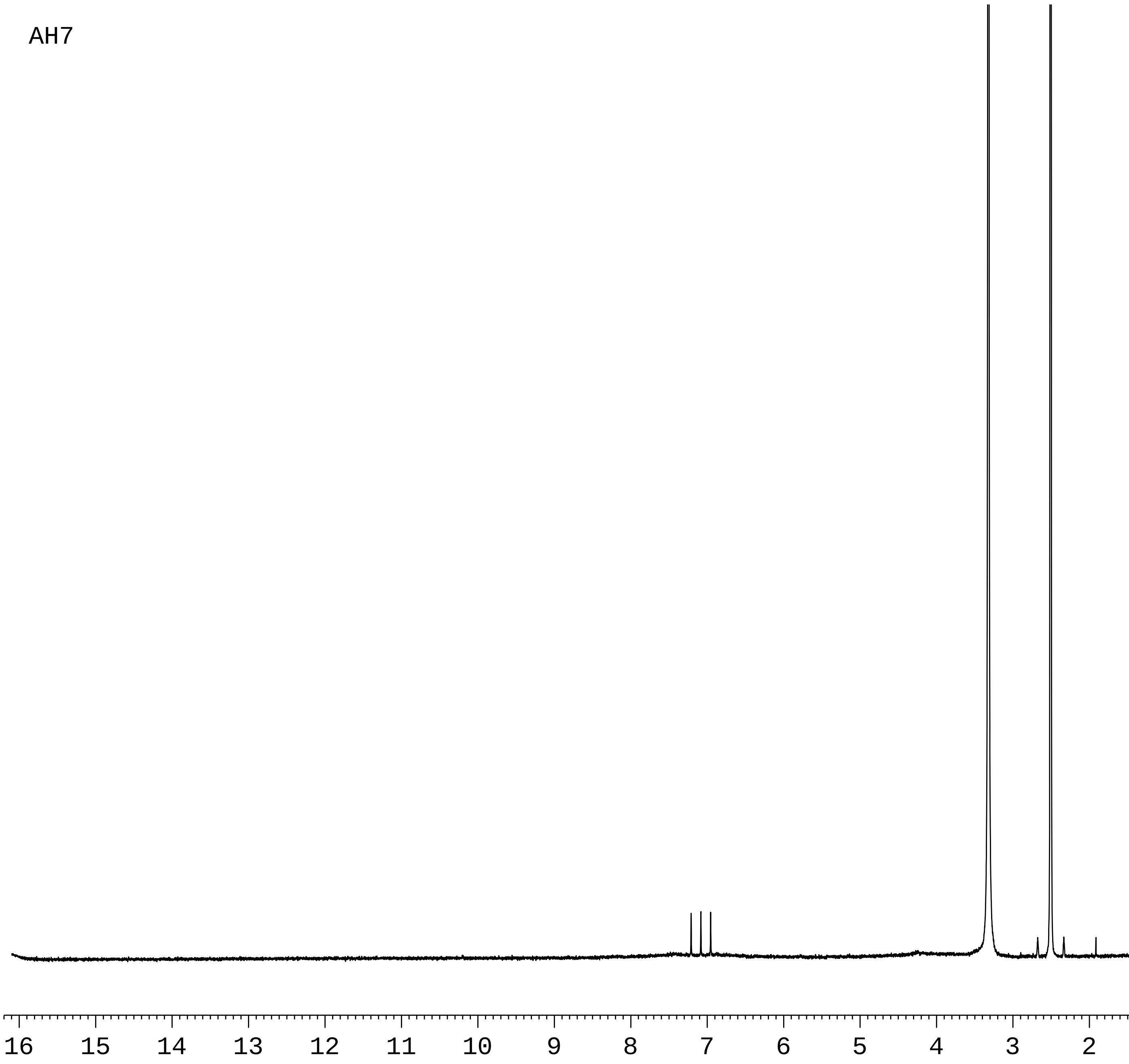
AH7
16
15
14
13
12
11
10
9
8
7
6
5
4
3
2
